# *N*′-(2-Hydr­oxy-3,5-diiodo­benzyl­idene)-2-methoxy­benzohydrazide

**DOI:** 10.1107/S160053680901914X

**Published:** 2009-05-29

**Authors:** San-Jun Peng, Fen Zhang

**Affiliations:** a College of Chemistry and Biological Engineering, Changsha University of Science and Technology, Changsha 410014, People’s Republic of China; b School of Foreign Languages, Jiangsu University, Zhenjiang 212013, People’s Republic of China

## Abstract

The title compound, C_15_H_12_I_2_N_2_O_3_, was synthesized by the condensation of equimolar amounts of 3,5-diiodo­salicylaldehyde and 2-methoxy­benzohydrazide in a methanol solution. There are two independent mol­ecules, *A* and *B*, in the asymmetric unit. The dihedral angle between the two benzene rings is 30.2 (2)° for mol­ecule *A* and 21.7 (2)° for mol­ecule B. There are intra­molecular O—H⋯N and N—H⋯O hydrogen bonds in each mol­ecule. The crystal studied was an inversion twin with a 0.59 (3):0.41 (3) domain ratio.

## Related literature

For background to Schiff bases and their complexes, see: Ali *et al.* (2005 ▶). For related structures, see: Yehye *et al.* (2008*a* ▶,*b* ▶); Jing *et al.* (2006 ▶); Ling *et al.* (2008 ▶).
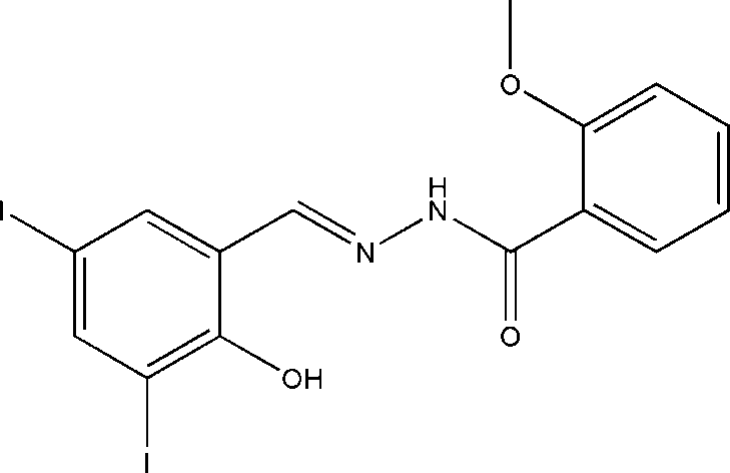


## Experimental

### 

#### Crystal data

C_15_H_12_I_2_N_2_O_3_
*M* *_r_* = 522.07
Orthorhombic, 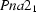

*a* = 16.073 (2) Å
*b* = 15.628 (2) Å
*c* = 13.284 (1) Å
*V* = 3336.8 (6) Å^3^
*Z* = 8
Mo *K*α radiation
μ = 3.78 mm^−1^
*T* = 298 K
0.23 × 0.20 × 0.20 mm

#### Data collection

Bruker SMART 1000 CCD area-detector diffractometer
Absorption correction: multi-scan (*SADABS*; Bruker, 2001 ▶) *T*_min_ = 0.432, *T*_max_ = 0.469
26178 measured reflections
7237 independent reflections
4902 reflections with *I* > 2σ(*I*)
*R*_int_ = 0.053

#### Refinement

*R*[*F* ^2^ > 2σ(*F* ^2^)] = 0.046
*wR*(*F* ^2^) = 0.101
*S* = 1.00
7237 reflections
407 parameters
3 restraints
H atoms treated by a mixture of independent and constrained refinement
Δρ_max_ = 0.78 e Å^−3^
Δρ_min_ = −0.56 e Å^−3^
Absolute structure: Flack (1983 ▶), 3436 Friedel pairs
Flack parameter: 0.59 (3)

### 

Data collection: *SMART* (Bruker, 2007 ▶); cell refinement: *SAINT* (Bruker, 2007 ▶); data reduction: *SAINT*; program(s) used to solve structure: *SHELXTL* (Sheldrick, 2008 ▶); program(s) used to refine structure: *SHELXTL*; molecular graphics: *SHELXTL*; software used to prepare material for publication: *SHELXTL*.

## Supplementary Material

Crystal structure: contains datablocks global, I. DOI: 10.1107/S160053680901914X/sj2624sup1.cif

Structure factors: contains datablocks I. DOI: 10.1107/S160053680901914X/sj2624Isup2.hkl

Additional supplementary materials:  crystallographic information; 3D view; checkCIF report

## Figures and Tables

**Table 1:** Hydrogen-bond geometry (Å, °)

*D*—H⋯*A*	*D*—H	H⋯*A*	*D*⋯*A*	*D*—H⋯*A*
O4—H4⋯N3	0.82	1.90	2.577 (8)	139
O1—H1⋯N1	0.82	1.92	2.568 (8)	136
N2—H2⋯O3	0.90 (3)	1.91 (6)	2.613 (8)	134 (8)
N4—H4*B*⋯O6	0.89 (5)	1.98 (7)	2.629 (9)	128 (7)

**Fig. 1.**
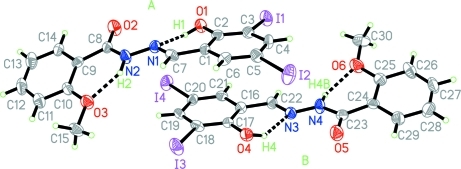
The molecular structure of (I), showing the atom-numbering scheme and 30% probability displacement ellipsoids. H atoms are shown as spheres of arbitrary radii.

## supplementary crystallographic information

### Comment

Schiff bases such as hydrazides are known to act as versatile ligands in coordination chemistry (Ali *et al.*, 2005). We report herein the crystal structure of the new title benzohydrazide derivative (I), Fig. 1.

Compound (I) consists two independent molecules, A and B in the asymmetric unit. The dihedral angles between the two benzene rings are 30.2 (2)° for A and 21.7 (2)° for B, respectively. All the bond lengths are comparable to those observed in other similar compounds (Yehye *et al.*, 2008a,b; Jing *et al.*, 2006); Ling *et al.*, 2008). There are two intramolecular O–H···N and N–H···O hydrogen bonds (Table 1) in each molecule.

### Experimental

2-Methoxybenzohydrazide (0.1 mmol, 16.6 mg) and 3,5-diiodosalicylaldehyde (0.1 mmol, 37.4 mg) were stirred at 318 K in methanol (10 ml) for 30 min. The filtrate was kept in air for a few days depositing colorless block-like crystals of (I).

### Refinement

The crystal studied was an inversion twin with a 0.59 (3):0.41 (3) domain ratio. The number of Friedel pairs in the data set is 3436. Atoms H2 and H4B were located in a difference Fourier map and refined isotropically, with the N–H distance restrained to 0.90 (1) Å, and with *U*_iso_ set to 0.08 Å^2^. All H atoms bound to carbon and oxygen were refined using riding models with d(C–H) = 0.93–0.96 Å, d(O–H) = 0.82 Å, *U*_iso_ = 1.2*U*_eq_(C) and 1.5*U*_eq_(O and methyl C).

### Crystal data

**Table:**

C_15_H_12_I_2_N_2_O_3_	*F*(000) = 1968
*M**_r_* = 522.07	*D*_x_ = 2.078 Mg m^−^^3^
Orthorhombic, *P**n**a*2_1_	Mo *K*α radiation, λ = 0.71073 Å
Hall symbol: P 2c -2n	Cell parameters from 4242 reflections
*a* = 16.073 (2) Å	θ = 2.3–24.5°
*b* = 15.628 (2) Å	µ = 3.78 mm^−^^1^
*c* = 13.284 (1) Å	*T* = 298 K
*V* = 3336.8 (6) Å^3^	Block, colorless
*Z* = 8	0.23 × 0.20 × 0.20 mm

### Data collection

**Table:**

Bruker SMART 1000 CCD area-detector diffractometer	7237 independent reflections
Radiation source: fine-focus sealed tube	4902 reflections with *I* > 2σ(*I*)
graphite	*R*_int_ = 0.053
ω scans	θ_max_ = 27.0°, θ_min_ = 1.8°
Absorption correction: multi-scan (*SADABS*; Bruker, 2001)	*h* = −20→20
*T*_min_ = 0.432, *T*_max_ = 0.469	*k* = −19→19
26178 measured reflections	*l* = −16→16

### Refinement

**Table:**

Refinement on *F*^2^	Secondary atom site location: difference Fourier map
Least-squares matrix: full	Hydrogen site location: inferred from neighbouring sites
*R*[*F*^2^ > 2σ(*F*^2^)] = 0.046	H atoms treated by a mixture of independent and constrained refinement
*wR*(*F*^2^) = 0.101	*w* = 1/[σ^2^(*F*_o_^2^) + (0.0412*P*)^2^] where *P* = (*F*_o_^2^ + 2*F*_c_^2^)/3
*S* = 1.00	(Δ/σ)_max_ < 0.001
7237 reflections	Δρ_max_ = 0.78 e Å^−^^3^
407 parameters	Δρ_min_ = −0.56 e Å^−^^3^
3 restraints	Absolute structure: Flack (1983), 3436 Friedel pairs
Primary atom site location: structure-invariant direct methods	Flack parameter: 0.59 (3)

### Special details

**Table:**

Geometry. All e.s.d.'s (except the e.s.d. in the dihedral angle between two l.s. planes) are estimated using the full covariance matrix. The cell e.s.d.'s are taken into account individually in the estimation of e.s.d.'s in distances, angles and torsion angles; correlations between e.s.d.'s in cell parameters are only used when they are defined by crystal symmetry. An approximate (isotropic) treatment of cell e.s.d.'s is used for estimating e.s.d.'s involving l.s. planes.
Refinement. Refinement of *F*^2^ against ALL reflections. The weighted *R*-factor *wR* and goodness of fit *S* are based on *F*^2^, conventional *R*-factors *R* are based on *F*, with *F* set to zero for negative *F*^2^. The threshold expression of *F*^2^ > σ(*F*^2^) is used only for calculating *R*-factors(gt) *etc*. and is not relevant to the choice of reflections for refinement. *R*-factors based on *F*^2^ are statistically about twice as large as those based on *F*, and *R*- factors based on ALL data will be even larger.

### Fractional atomic coordinates and isotropic or equivalent isotropic displacement parameters (Å^2^)

**Table:**

	*x*	*y*	*z*	*U*_iso_*/*U*_eq_	
I3	0.40540 (3)	0.37109 (4)	0.59648 (6)	0.0789 (2)	
I4	0.75672 (4)	0.29448 (4)	0.71249 (6)	0.0744 (2)	
O4	0.4652 (3)	0.5530 (4)	0.6664 (5)	0.0592 (16)	
H4	0.4752	0.5974	0.6971	0.071*	
O5	0.4563 (4)	0.7984 (3)	0.7103 (6)	0.0738 (18)	
O6	0.7003 (4)	0.8732 (4)	0.7109 (6)	0.0745 (19)	
N3	0.5663 (4)	0.6711 (4)	0.7246 (6)	0.0492 (17)	
N4	0.5871 (4)	0.7542 (4)	0.7391 (6)	0.0552 (19)	
C16	0.6078 (4)	0.5270 (5)	0.7135 (7)	0.0428 (18)	
C17	0.5299 (5)	0.4972 (5)	0.6787 (6)	0.0430 (19)	
C18	0.5183 (5)	0.4121 (5)	0.6577 (7)	0.052 (2)	
C19	0.5820 (5)	0.3546 (5)	0.6660 (6)	0.048 (2)	
H19	0.5740	0.2974	0.6493	0.057*	
C20	0.6582 (5)	0.3831 (5)	0.6994 (6)	0.047 (2)	
C21	0.6718 (5)	0.4676 (5)	0.7198 (7)	0.053 (2)	
H21	0.7248	0.4856	0.7381	0.064*	
C22	0.6248 (5)	0.6155 (5)	0.7341 (6)	0.047 (2)	
H22	0.6777	0.6325	0.7541	0.057*	
C23	0.5297 (5)	0.8171 (5)	0.7244 (6)	0.0452 (19)	
C24	0.5589 (5)	0.9062 (5)	0.7306 (6)	0.0455 (19)	
C25	0.6422 (5)	0.9340 (5)	0.7234 (7)	0.053 (2)	
C26	0.6607 (7)	1.0209 (6)	0.7251 (8)	0.074 (3)	
H26	0.7154	1.0391	0.7171	0.089*	
C27	0.6000 (9)	1.0786 (6)	0.7381 (7)	0.085 (4)	
H27	0.6137	1.1362	0.7431	0.102*	
C28	0.5176 (8)	1.0543 (7)	0.7445 (7)	0.083 (3)	
H28	0.4758	1.0950	0.7518	0.100*	
C29	0.4986 (6)	0.9687 (6)	0.7397 (6)	0.061 (2)	
H29	0.4430	0.9522	0.7428	0.073*	
C30	0.7849 (5)	0.8953 (7)	0.6937 (8)	0.080 (3)	
H30A	0.8055	0.9276	0.7499	0.119*	
H30B	0.7890	0.9292	0.6336	0.119*	
H30C	0.8173	0.8441	0.6860	0.119*	
I1	0.81885 (3)	0.63308 (4)	0.55553 (6)	0.0747 (2)	
I2	0.46921 (4)	0.71070 (4)	0.43332 (6)	0.0793 (2)	
O1	0.7606 (3)	0.4525 (4)	0.4793 (5)	0.0549 (16)	
H1	0.7425	0.4041	0.4890	0.082*	
O2	0.7669 (3)	0.2033 (4)	0.4301 (7)	0.076 (2)	
O3	0.5224 (3)	0.1360 (3)	0.4748 (5)	0.0639 (16)	
N1	0.6578 (4)	0.3339 (4)	0.4300 (5)	0.0465 (16)	
N2	0.6348 (4)	0.2499 (4)	0.4275 (6)	0.0540 (17)	
C1	0.6177 (4)	0.4794 (5)	0.4342 (6)	0.0407 (18)	
C2	0.6962 (5)	0.5073 (5)	0.4661 (6)	0.046 (2)	
C3	0.7075 (4)	0.5914 (5)	0.4888 (6)	0.048 (2)	
C4	0.6443 (5)	0.6517 (5)	0.4757 (6)	0.052 (2)	
H4A	0.6537	0.7094	0.4883	0.063*	
C5	0.5680 (5)	0.6235 (5)	0.4438 (7)	0.053 (2)	
C6	0.5548 (5)	0.5392 (5)	0.4207 (6)	0.0451 (19)	
H6	0.5035	0.5219	0.3958	0.054*	
C7	0.6016 (5)	0.3892 (5)	0.4185 (7)	0.048 (2)	
H7	0.5486	0.3717	0.3996	0.057*	
C8	0.6933 (5)	0.1877 (5)	0.4350 (7)	0.0462 (19)	
C9	0.6631 (5)	0.0984 (5)	0.4467 (6)	0.044 (2)	
C10	0.5797 (5)	0.0735 (5)	0.4667 (6)	0.047 (2)	
C11	0.5616 (6)	−0.0131 (6)	0.4790 (7)	0.063 (2)	
H11	0.5075	−0.0300	0.4938	0.075*	
C12	0.6220 (8)	−0.0729 (6)	0.4696 (8)	0.077 (3)	
H12	0.6084	−0.1304	0.4766	0.093*	
C13	0.7045 (7)	−0.0501 (6)	0.4495 (7)	0.074 (3)	
H13	0.7460	−0.0913	0.4437	0.089*	
C14	0.7219 (6)	0.0346 (5)	0.4387 (6)	0.052 (2)	
H14	0.7766	0.0503	0.4252	0.062*	
C15	0.4365 (5)	0.1115 (6)	0.4888 (7)	0.066 (3)	
H15A	0.4293	0.0880	0.5550	0.099*	
H15B	0.4215	0.0694	0.4394	0.099*	
H15C	0.4015	0.1609	0.4813	0.099*	
H2	0.5807 (15)	0.237 (5)	0.435 (7)	0.080*	
H4B	0.639 (2)	0.770 (5)	0.754 (7)	0.080*	

### Atomic displacement parameters (Å^2^)

**Table:**

	*U*^11^	*U*^22^	*U*^33^	*U*^12^	*U*^13^	*U*^23^
I3	0.0459 (3)	0.0612 (4)	0.1295 (6)	−0.0160 (3)	−0.0114 (4)	−0.0033 (4)
I4	0.0618 (4)	0.0584 (4)	0.1029 (5)	0.0198 (3)	−0.0071 (4)	−0.0048 (4)
O4	0.034 (3)	0.054 (3)	0.089 (5)	−0.002 (3)	0.003 (3)	−0.001 (3)
O5	0.056 (4)	0.053 (4)	0.112 (5)	0.002 (3)	0.003 (4)	0.002 (4)
O6	0.048 (4)	0.067 (4)	0.108 (6)	−0.011 (3)	0.003 (4)	0.010 (4)
N3	0.041 (4)	0.044 (4)	0.063 (5)	−0.004 (3)	0.002 (4)	−0.006 (4)
N4	0.045 (4)	0.038 (4)	0.083 (6)	−0.001 (3)	−0.012 (4)	−0.012 (4)
C16	0.029 (4)	0.043 (4)	0.056 (5)	0.008 (3)	−0.007 (4)	0.006 (4)
C17	0.045 (5)	0.042 (4)	0.042 (5)	−0.003 (4)	0.009 (4)	−0.003 (4)
C18	0.038 (4)	0.056 (5)	0.060 (6)	−0.006 (4)	0.001 (4)	0.016 (5)
C19	0.057 (5)	0.040 (4)	0.047 (5)	−0.009 (4)	0.009 (4)	0.008 (4)
C20	0.046 (4)	0.045 (5)	0.051 (5)	0.009 (4)	0.003 (4)	−0.002 (4)
C21	0.039 (4)	0.056 (5)	0.064 (6)	−0.009 (4)	0.013 (4)	−0.005 (5)
C22	0.046 (5)	0.043 (5)	0.053 (6)	−0.009 (4)	−0.005 (4)	−0.009 (4)
C23	0.042 (5)	0.053 (5)	0.041 (5)	−0.006 (4)	0.004 (4)	−0.003 (4)
C24	0.047 (4)	0.050 (5)	0.039 (5)	−0.001 (4)	0.007 (4)	0.006 (4)
C25	0.056 (5)	0.054 (5)	0.048 (5)	−0.002 (4)	−0.005 (4)	0.006 (4)
C26	0.093 (7)	0.053 (6)	0.076 (7)	−0.026 (6)	−0.015 (6)	0.008 (6)
C27	0.152 (12)	0.038 (5)	0.065 (7)	−0.015 (7)	0.019 (7)	0.002 (5)
C28	0.111 (9)	0.064 (7)	0.075 (8)	0.026 (6)	0.023 (7)	0.003 (5)
C29	0.079 (6)	0.056 (6)	0.047 (6)	−0.003 (5)	0.005 (5)	0.003 (4)
C30	0.055 (6)	0.115 (8)	0.069 (7)	−0.009 (6)	0.000 (5)	0.016 (6)
I1	0.0441 (3)	0.0585 (4)	0.1215 (6)	−0.0146 (3)	−0.0043 (3)	−0.0101 (4)
I2	0.0761 (4)	0.0635 (4)	0.0985 (5)	0.0285 (3)	−0.0265 (4)	−0.0121 (4)
O1	0.031 (3)	0.045 (3)	0.088 (5)	0.004 (2)	−0.002 (3)	0.000 (3)
O2	0.033 (3)	0.058 (4)	0.137 (6)	0.003 (3)	0.002 (4)	−0.004 (4)
O3	0.045 (3)	0.051 (3)	0.096 (5)	−0.003 (3)	0.005 (3)	0.000 (3)
N1	0.039 (4)	0.042 (4)	0.058 (4)	0.001 (3)	−0.001 (3)	−0.007 (4)
N2	0.041 (4)	0.039 (4)	0.082 (5)	−0.007 (3)	−0.004 (4)	−0.003 (4)
C1	0.039 (4)	0.037 (4)	0.047 (5)	−0.002 (3)	0.003 (4)	−0.007 (4)
C2	0.035 (4)	0.050 (5)	0.052 (5)	0.002 (4)	0.007 (4)	0.005 (4)
C3	0.038 (4)	0.051 (5)	0.053 (5)	−0.006 (4)	0.001 (4)	−0.005 (4)
C4	0.059 (6)	0.033 (4)	0.065 (6)	0.001 (4)	−0.004 (5)	−0.009 (4)
C5	0.055 (5)	0.048 (5)	0.057 (6)	0.003 (4)	−0.004 (4)	0.008 (5)
C6	0.040 (4)	0.061 (5)	0.035 (5)	0.008 (4)	−0.013 (4)	−0.004 (4)
C7	0.035 (4)	0.056 (5)	0.053 (5)	−0.012 (4)	−0.005 (4)	0.007 (4)
C8	0.043 (5)	0.037 (4)	0.058 (5)	0.002 (4)	0.007 (4)	−0.008 (4)
C9	0.052 (5)	0.045 (4)	0.034 (5)	0.008 (4)	0.005 (4)	0.000 (4)
C10	0.062 (5)	0.043 (5)	0.035 (5)	−0.013 (4)	−0.007 (4)	0.003 (4)
C11	0.070 (6)	0.053 (6)	0.064 (6)	−0.014 (5)	−0.014 (5)	0.001 (5)
C12	0.127 (10)	0.038 (5)	0.068 (7)	−0.007 (6)	−0.009 (6)	−0.002 (5)
C13	0.112 (9)	0.043 (5)	0.068 (7)	0.002 (6)	−0.018 (6)	0.001 (5)
C14	0.061 (5)	0.043 (5)	0.051 (5)	0.004 (4)	−0.001 (5)	−0.008 (4)
C15	0.054 (5)	0.080 (6)	0.065 (6)	−0.029 (5)	−0.002 (5)	−0.002 (5)

### Geometric parameters (Å, °)

**Table:**

I3—C18	2.090 (8)	I1—C3	2.101 (7)
I4—C20	2.111 (7)	I2—C5	2.098 (8)
O4—C17	1.367 (9)	O1—C2	1.355 (8)
O4—H4	0.8200	O1—H1	0.8200
O5—C23	1.229 (9)	O2—C8	1.210 (9)
O6—C25	1.342 (10)	O3—C10	1.348 (10)
O6—C30	1.421 (10)	O3—C15	1.445 (9)
N3—C22	1.287 (10)	N1—C7	1.259 (10)
N3—N4	1.354 (8)	N1—N2	1.364 (8)
N4—C23	1.362 (10)	N2—C8	1.356 (9)
N4—H4B	0.89 (5)	N2—H2	0.90 (3)
C16—C21	1.389 (10)	C1—C6	1.389 (10)
C16—C17	1.414 (10)	C1—C2	1.400 (10)
C16—C22	1.437 (10)	C1—C7	1.448 (10)
C17—C18	1.371 (11)	C2—C3	1.360 (10)
C18—C19	1.366 (11)	C3—C4	1.396 (11)
C19—C20	1.377 (11)	C4—C5	1.370 (11)
C19—H19	0.9300	C4—H4A	0.9300
C20—C21	1.365 (10)	C5—C6	1.369 (10)
C21—H21	0.9300	C6—H6	0.9300
C22—H22	0.9300	C7—H7	0.9300
C23—C24	1.472 (11)	C8—C9	1.485 (11)
C24—C29	1.382 (12)	C9—C14	1.379 (10)
C24—C25	1.412 (11)	C9—C10	1.421 (11)
C25—C26	1.390 (12)	C10—C11	1.393 (11)
C26—C27	1.339 (15)	C11—C12	1.353 (13)
C26—H26	0.9300	C11—H11	0.9300
C27—C28	1.381 (15)	C12—C13	1.399 (14)
C27—H27	0.9300	C12—H12	0.9300
C28—C29	1.374 (12)	C13—C14	1.360 (12)
C28—H28	0.9300	C13—H13	0.9300
C29—H29	0.9300	C14—H14	0.9300
C30—H30A	0.9600	C15—H15A	0.9600
C30—H30B	0.9600	C15—H15B	0.9600
C30—H30C	0.9600	C15—H15C	0.9600
			
C17—O4—H4	109.4	C2—O1—H1	109.5
C25—O6—C30	120.9 (7)	C10—O3—C15	118.1 (6)
C22—N3—N4	116.9 (7)	C7—N1—N2	117.7 (7)
N3—N4—C23	120.3 (7)	C8—N2—N1	120.1 (6)
N3—N4—H4B	121 (6)	C8—N2—H2	120 (6)
C23—N4—H4B	118 (6)	N1—N2—H2	118 (6)
C21—C16—C17	117.1 (7)	C6—C1—C2	119.0 (7)
C21—C16—C22	119.4 (7)	C6—C1—C7	120.5 (7)
C17—C16—C22	123.3 (7)	C2—C1—C7	120.5 (7)
O4—C17—C18	119.5 (7)	O1—C2—C3	118.7 (7)
O4—C17—C16	120.1 (7)	O1—C2—C1	122.0 (7)
C18—C17—C16	120.4 (7)	C3—C2—C1	119.2 (7)
C19—C18—C17	121.3 (8)	C2—C3—C4	121.8 (7)
C19—C18—I3	118.7 (7)	C2—C3—I1	120.4 (6)
C17—C18—I3	119.6 (6)	C4—C3—I1	117.6 (6)
C18—C19—C20	118.7 (8)	C5—C4—C3	118.2 (8)
C18—C19—H19	120.7	C5—C4—H4A	120.9
C20—C19—H19	120.7	C3—C4—H4A	120.9
C21—C20—C19	121.3 (8)	C6—C5—C4	121.2 (8)
C21—C20—I4	119.9 (6)	C6—C5—I2	119.5 (6)
C19—C20—I4	118.8 (6)	C4—C5—I2	119.3 (6)
C20—C21—C16	121.0 (8)	C5—C6—C1	120.4 (7)
C20—C21—H21	119.5	C5—C6—H6	119.8
C16—C21—H21	119.5	C1—C6—H6	119.8
N3—C22—C16	119.5 (7)	N1—C7—C1	121.5 (8)
N3—C22—H22	120.3	N1—C7—H7	119.2
C16—C22—H22	120.3	C1—C7—H7	119.2
O5—C23—N4	120.1 (7)	O2—C8—N2	121.9 (7)
O5—C23—C24	122.6 (7)	O2—C8—C9	121.0 (7)
N4—C23—C24	117.3 (7)	N2—C8—C9	117.1 (7)
C29—C24—C25	117.0 (8)	C14—C9—C10	117.6 (8)
C29—C24—C23	116.7 (7)	C14—C9—C8	116.6 (7)
C25—C24—C23	126.2 (7)	C10—C9—C8	125.9 (7)
O6—C25—C26	123.0 (8)	O3—C10—C11	123.5 (8)
O6—C25—C24	116.7 (7)	O3—C10—C9	117.4 (7)
C26—C25—C24	120.2 (9)	C11—C10—C9	119.0 (8)
C27—C26—C25	120.3 (10)	C12—C11—C10	120.7 (9)
C27—C26—H26	119.9	C12—C11—H11	119.7
C25—C26—H26	119.9	C10—C11—H11	119.7
C26—C27—C28	121.5 (9)	C11—C12—C13	121.5 (9)
C26—C27—H27	119.3	C11—C12—H12	119.3
C28—C27—H27	119.3	C13—C12—H12	119.3
C29—C28—C27	118.6 (10)	C14—C13—C12	117.5 (10)
C29—C28—H28	120.7	C14—C13—H13	121.2
C27—C28—H28	120.7	C12—C13—H13	121.2
C28—C29—C24	122.4 (9)	C13—C14—C9	123.7 (9)
C28—C29—H29	118.8	C13—C14—H14	118.1
C24—C29—H29	118.8	C9—C14—H14	118.1
O6—C30—H30A	109.5	O3—C15—H15A	109.5
O6—C30—H30B	109.5	O3—C15—H15B	109.5
H30A—C30—H30B	109.5	H15A—C15—H15B	109.5
O6—C30—H30C	109.5	O3—C15—H15C	109.5
H30A—C30—H30C	109.5	H15A—C15—H15C	109.5
H30B—C30—H30C	109.5	H15B—C15—H15C	109.5

### Hydrogen-bond geometry (Å, °)

**Table:**

*D*—H···*A*	*D*—H	H···*A*	*D*···*A*	*D*—H···*A*
O4—H4···N3	0.82	1.90	2.577 (8)	139
O1—H1···N1	0.82	1.92	2.568 (8)	136
N2—H2···O3	0.90 (3)	1.91 (6)	2.613 (8)	134 (8)
N4—H4B···O6	0.89 (5)	1.98 (7)	2.629 (9)	128 (7)
